# From Misperception to Prevention: Improving Cardiovascular Health and Risk Perception Through Risk Communication in Hungary

**DOI:** 10.3390/healthcare14091229

**Published:** 2026-05-03

**Authors:** Blanka Ehrenberger, Orsolya Papp-Zipernovszky, Alexandra Assabiny, József Otohal, Gergely Koplányi, Béla Merkely, Zsolt Bagyura, Márta Csabai, Zsófia Ocsovszky

**Affiliations:** 1 Heart and Vascular Center, Semmelweis University, 1122 Budapest, Hungary; papp.zipernovszky.orsolya@semmelweis.hu (O.P.-Z.); assabiny.alexandra@semmelweis.hu (A.A.); otohal.jozsef@semmelweis.hu (J.O.); koplanyi@okri.hu (G.K.); merkely.bela@semmelweis.hu (B.M.); bagyura.zsolt@semmelweis.hu (Z.B.); csabai.marta@kre.hu (M.C.); ocsovszky.zsofia@semmelweis.hu (Z.O.); 2 Department of Personality and Health Psychology, Institute of Psychology, Eötvös Loránd University, 1064 Budapest, Hungary; 3 Department of Personality and Health Psychology, Institute of Psychology, Károli Gáspár University of the Reformed Church, 1037 Budapest, Hungary

**Keywords:** risk estimation accuracy, risk perception gap, cardiovascular risk communication

## Abstract

Background/Objectives: Effective cardiovascular prevention requires improved risk perception and appropriate communication strategies that support cost-effective interventions. This study evaluated one-year changes in cardiovascular risk estimation accuracy and examined associations between communication strategies, accuracy, and health outcomes. Methods: We analyzed 200 participants (mean age: 56.06 ± 6.26; 32.2% male) in a population-based study conducted in Hungary. Cardiovascular risk was calculated using the Framingham Risk Score based on laboratory and anthropometric measures, and subjective risk perception was assessed and categorized as realistic, optimistic, or pessimistic relative to objective risk. All participants received written risk feedback, while subgroups additionally participated in individual or group-based risk communication. Results: After 12 months, the proportion of participants with accurate risk perception increased from 39.0% to 50.5% (*p* = 0.012), accompanied by a significant reduction in pessimistic estimations (*p* = 0.013). We could not observe significant differences in estimation accuracy between communication strategies. The written-only communication group showed a significant decrease in cardiovascular risk factors (weight (*p* = 0.014), BMI (*p* = 0.042), blood pressure (*p* = 0.035), and LDL levels (*p* = 0.001)). No significant differences were found in health outcomes between risk communication groups. Conclusions: These results demonstrate that even written-only communication may be an effective way to improve cardiovascular health outcomes, possibly by correcting risk perception gaps, suggesting that cost-effective, low-intensity communication strategies may be sufficient to support primary prevention efforts.

## 1. Introduction

According to the 2023 statistics from the European Society of Cardiology, the mortality rate from cardiovascular diseases (CVDs) has decreased by more than 50% in ESC member countries. However, it remains the leading cause of death, accounting for more than 1.6 million deaths among women and 1.5 million deaths among men each year [1]. This places a substantial burden on the global economy and healthcare system. Heart and vascular diseases, such as ischemic heart disease and stroke, were responsible for 40% of all registered deaths in Hungary in 2021 [2]. The healthcare system needs evidence-based, cost-effective preventive methods to help providers allocate their resources more effectively and reduce burdens.

A substantial portion of CVD is preventable through the management of behavioral risk factors; therefore, prevention and intervention efforts should focus on promoting health-seeking behaviors within the population.

### 1.1. Risk Perception Gap

Understanding patients’ own risk plays a crucial role in decision-making related to adopting healthier behaviors and preventing CVD [3]. According to the Health Belief Model (HBM) [4], accurate assessment of health risks increases perceived vulnerability, which in turn motivates individuals to engage in health-seeking behavior. The model suggests that subjective CVD risk depends on perceived vulnerability, threat awareness, societal influences, and the perceived benefits of preventive actions. When individuals under- or overestimate their personal risk, their perceived vulnerability may no longer align with their actual risk, potentially leading to inadequate or unnecessary preventive behaviors.

Multiple studies [5,6] have highlighted a gap between perceived and calculated CVD risks, often revealing excessive optimism [7]. A comparative study [8] confirmed that many individuals underestimated their heart attack and stroke risks, even in the presence of apparent risk factors. Individuals who tend to under- or overestimate their risk often perceive risk in a binary way rather than as a continuum. In addition, when assessing their risk, they compare themselves to individuals with more severe conditions rather than average peers with similar characteristics [7].

These findings underscore the need for targeted interventions to improve patients’ understanding of CVD risks and support informed preventive decision-making.

### 1.2. Risk Communication and Its Challenges

Risk communication, as defined by the World Health Organization (WHO), involves the continuous exchange of information between experts and individuals facing threats to their health or well-being. Its aim is to empower individuals to make informed decisions and adopt protective measures [9]. In cardiovascular (CV) health, effective communication supports the management of CVD risks through lifestyle changes and medications, while respecting patient preferences and fostering collaboration. Effective communication requires understanding patients’ health literacy and social background, with messages tailored accordingly [10].

The 2021 European Society of Cardiology (ESC) Guideline [11] recommends a stepwise approach to risk management, emphasizing that risk communication should begin with lifestyle advice for all patients, regardless of risk level. The second step should be individualized and integrated into a shared decision-making process, considering patient preferences, comorbidities, and treatment goals. The guideline underscores the importance of ensuring whether patients fully comprehend their health risks, identify the specific risk factors that should be mitigated in their case, and are aware of the benefits and drawbacks of interventions aimed at mitigating these factors.

Evidence underscores the importance of assessing absolute CV risk alongside key risk factors for effective CVD management [7]. International guidelines advocate using CV risk assessment tools to estimate 5- or 10-year risk. Despite recommendations, CV risk assessment tools are underutilized in primary care, with usage rates ranging from 17% to 47% [12,13,14]. Studies reveal that general practitioners rarely conduct risk assessments, citing barriers such as confusion between absolute and relative risk, poor integration of tools, and limited practical knowledge [15,16]. One key reason for underutilization is the perceived inaccuracy of these tools: studies have shown considerable variability across calculators, sometimes leading to inconsistent risk categorization for the same patient [17]. Moreover, clinicians often consider a broader range of contextual and patient-specific factors when making therapeutic decisions, than those captured by formal tools [18]. Evidence suggests that these tools can be valuable in patient communication [19]. Besides absolute risk estimation, effective risk communication can enhance clinical outcomes, particularly in combination with other behavioral strategies [20]. Risk communication can improve patients’ understanding of CV risk and support shared decision-making when used appropriately.

According to the literature, CV risk communication, regardless of the strategy employed, reduces risk factors and improves patients’ subjective perceptions. A meta-analysis of 62 randomized controlled trials found that patients who participated in risk communication had significantly better understanding of CV risk and experienced a reduction in risk after 12 months of follow-up compared to those who received usual care [21].

While risk communication is key to prevention, data comparing different risk communication methods aimed at bridging the gap between subjective and objective CVD risk and their long-term impact on risk perception accuracy remain limited. In particular, few studies have investigated whether risk communication can effectively correct systematic risk misperceptions over time, especially in Central and Eastern Europe. To address this gap this study aimed to evaluate changes in the accuracy of CV risk estimation over one year period following risk assessment and communication, and to examine whether different communication strategies were associated with improved estimation accuracy and objective health outcomes. We hypothesized that, over the one-year period, the proportion of accurate estimators (realistic risk perceivers) would increase significantly depending on the type of risk communication (written-only, written + individual, written + group), whereas the proportions of overestimators (pessimistic perceivers) and underestimators (optimistic perceivers) would decrease. Furthermore, we sought to determine whether written-only risk communication alone could be sufficient to enhance estimation accuracy and promote measurable improvements in CV health indicators between 2023 and 2024.

## 2. Materials and Methods

### 2.1. Study Design

The Budakalász Epidemiological Study [22] was initiated in 2012 to investigate the complex cardiovascular health of the population of Budakalász, a suburban settlement near Budapest, Hungary, enrolling 2420 participants. To continue the research, we subsequently extended the study. The current research phase of the Extended Budakalász Study was conducted between 2023 and 2024. In this phase, the original participants from the 2012 screening were reinvited, and the study population was expanded to include residents of the 9th district of Budapest as well employees of Semmelweis University. No data from 2012 were used in this analysis.

After a baseline assessment of participants’ general health in 2023 (medical measures for CVD risk calculations, lifestyle, and psychological measures) participants received written feedback electronically via password-protected email attachments and in printed form during individual and group sessions (Document S1 Supplementary Material) about their calculated CV risk (Framingham Score and Score2) and cardioprotective lifestyle recommendations.

Participants were assigned to one of the three risk communication groups using a systematic allocation method based on their chronological order of entry in the central Biobank database (1:1:1 ratio). To minimize selection bias, we conducted the assignment retrospectively after all screening days had been completed. This approach ensured that both participants and clinical staff remained blinded to group allocation throughout data collection. The cyclical nature of the assignment process (groups 1-2-3, repeated) ensured that participants arriving at different times of the day or on different screening days had an equal probability of being assigned to any group, thereby maintaining temporal balance. Furthermore, multiple simultaneous screening points and variations in individual data entry times introduced a practical element of randomness to the final database sequence, further mitigating the risk of systematic bias.

Accordingly, participants were allocated to Group 1 (written-only), Group 2 (written + individual risk communication), or Group 3 (written + group risk communication).

Subsequently, participants were offered the opportunity to join intervention groups (exercises, lifestyle club, dietetic group) aimed at facilitating long-term health behavior changes over a 12 month period at no cost. In 2024, all available participants were followed up and underwent the same assessments as one year earlier.

### 2.2. Sample and Procedures

In 2023, participants were recruited from the 9th district of Budapest, the agglomeration of Budapest (Budakalász), and employees of Semmelweis University. Participants (*n* = 376) were recruited by general practitioners. Inclusion criteria targeted individuals aged 45–65 years with a laboratory report from the preceding four months, including key cardiometabolic parameters (cholesterol, triglycerides, LDL, HDL, blood glucose, HbA1c). Exclusion criteria included severe CVDs, diabetes with organ damage, certain chronic non-communicable diseases, and any condition that, in the judgment of the attending physician, would prevent active participation in the program.

They received both written and verbal information about the study and provided written informed consent form. Blood samples and physical examinations were conducted (including measurements of blood pressure, weight, Body Mass Index (BMI), and InBody analysis). A questionnaire was completed in two sessions: one administered by an examiner during the examination and the other self-completed prior to the visit. The questionnaires assessed subjective health status, CV risk, psychological well-being, health-related behaviors, and demographic characteristics.

Written feedback was provided as described above.

In 2024, we conducted a follow-up study using the same measurements (*n* = 213). Due to missing laboratory data, the Framingham Risk Scores could not be calculated for 13 participants; therefore, after data cleaning, the current analysis included data of 200 participants.

In terms of the perception gap, participants were categorized into three groups: “realistic” (accurate estimators); “optimistic” (underestimators of their actual risk); and “pessimistic” (overestimators of their objective CV risk). Group classification was based on the concordance or discrepancy between objective and perceived risk.

### 2.3. Risk Communication Strategies Applied

#### 2.3.1. Written-Only Risk Communication

According to our written risk communication protocol, each participant received a detailed feedback report summarizing their laboratory results and CV risk profile based on the Framingham and SCORE2 scales. Individual risk was classified as low, moderate, or high, accompanied by exact percentage values, brief explanatory notes, and a concise summary of psychological status (see Document S1). In 2024, the feedback also highlighted changes in each participant’s risk level from 2023, indicating whether their condition had improved, worsened, or remained stable. The laboratory section included lipid profile, blood glucose, HbA1c, blood pressure, BMI, and type 2 diabetes risk, with each value classified as normal, elevated, or abnormal. Psychological assessments provided narrative feedback on mood and perceived stress, along with recommendations to seek professional support if required. The 2024 reports also included tailored recommendations for CV risk reduction, with a focus on SMART goal-setting and stress management strategies to support long-term health improvement.

#### 2.3.2. In-Person Individual Risk Communication

The individual risk communication protocol aimed to enhance participants’ understanding of their own CVD risks and encourage health behavior changes. Each individual session lasted approximately 50–55 min and was conducted by trained psychologists (health psychologists and clinical psychology residents).

The protocol was structured into several key phases. First, the participants’ baseline CVD knowledge and subjective risk perception were assessed. This was followed by a brief educational session on modifiable risk factors and the Framingham and SCORE2 objective risk assessment models. To facilitate the understanding of numerical risk data, psychologists used visual aids, such as three-colored (green—low risk, yellow—medium risk, red—high risk) scales without numerical labels and a 100-person pictogram (where participants estimated the number of “at-risk” individuals). These interactive tasks helped identify misperceptions of risks, such as under- or overestimation (e.g., due to optimistic bias).

The second half of the intervention focused on behavior change using a specialized Planning Workbook. Psychologists addressed the participants’ self-efficacy and identified specific barriers to change, such as chronic stress or time management issues. The session concluded with the development of a personalized health behavior change plan based on SMART goals, and aimed at either initiating new health behaviors or reinforcing existing ones. Participants also completed a feedback questionnaire evaluating the session.

#### 2.3.3. In-Person Group Risk Communication

The group risk communication protocol followed a structured 120 min format and was also conducted by trained psychologists in groups of approximately 30 participants, further subdivided into smaller, heterogeneous (low, middle, and high risk) units of 5–6 individuals. The session began with participants discussing their baseline knowledge of CVD risks and their personal motivations. Through interactive “post-it” brainstorming sessions using flipcharts, participants identified CVD risk factors and categorized them into modifiable and non-modifiable groups.

The group leader then introduced the Framingham and SCORE 2 risk assessment models. To bridge the gap between subjective and objective risks, participants engaged in an exercise where they physically positioned themselves on colored markers (green, yellow, red) corresponding to their perceived risk categories. This was accompanied by visual aids, such as a 100-person pictogram, to clarify the statistical meaning of their risk scores. The protocol addressed risk misperception through group discussions and a psychodramatic reflection on personal health status.

The final phase of the intervention focused on self-efficacy and behavioral change. Participants moved into specialized “thematic stations” (e.g., nutrition, physical activity, stress management) according to their primary risk factors. Here, they collaborated to identify barriers and develop implementation strategies, which were recorded in a Planning Workbook. The session, similar to the individual risk communication, concluded with a feedback questionnaire, the formulation of concrete, personalized action plans (SMART goals), and a final assessment of their motivation to change.

**Figure 1:**
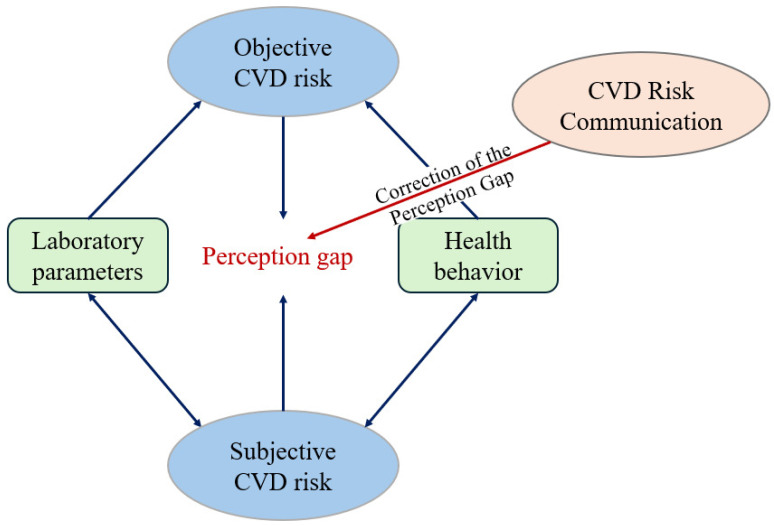
Conceptualization of our risk communication strategy.

The conceptualization of risk communication aimed at bridging the risk perception gap is presented in Figure 1.

### 2.4. Measures

#### 2.4.1. Framingham Risk Score (FRS)

In the study, we used the Framingham Risk Score to estimate participants’ objective CV risk. The reason for using the Framingham Risk Score was, on the one hand, that the study employed it from the beginning. On the other hand, by evidence of the literature, this tool provides a clearer, more intuitive understanding, thereby demonstrating higher communicability [19,23,24]. The Framingham Risk Score uses traditional risk factors to estimate 10-year CV risk, encompassing events such as stroke, heart failure, and myocardial infarction. Participants were divided into three groups based on their risk: low (<10%), moderate (10–20%), and high (>20%) [19].

#### 2.4.2. CVD Subjective Risk Perception (SRP)

We used a single-item tool to assess subjective CVD risk. Given that perceived risk is fundamentally a global assessment rather than a complex construct, using a single item remains a standard practice in the field of cardiovascular health [25,26,27]. Participants were asked “What do you think is the likelihood that you will experience CVDs in the next 10 years?”, to which they could respond by choosing from the following options: “low,” “medium,” and “high” [28].

### 2.5. Statistical Analysis

We created dichotomous variables to statistically test changes in membership frequency in the estimation group between 2023 and 2024. We used the McNemar test to determine whether the frequency differences were significant. The Chi-square test was used to determine whether the type of risk communication affected estimation accuracy. We applied the Wilcoxon signed rank test to examine which medical factors changed significantly in the risk communication groups (written-only, individual, and group) between 2023 and 2024.

## 3. Results

### 3.1. Participant Characteristics

Data from 200 participants were analyzed in our study. The average age was 56.06 ± 6.26 years. Of the participants, 64 (32.2%) were male, and 135 (67.8%) were female.

In terms of employment status, 165 (85.9%) individuals were actively employed, 27 (13.6%) were retired, and 7 (3.5%) were unemployed or reported another category.

In terms of marital status, 148 participants (74.4%) were married, 6 (3%) were married but living separately, 15 (7.6%) were single, 11 (5.6%) were widowed, and 18 (9.1%) were divorced. Two participants did not respond to this question.

As for educational attainment, 137 respondents (69.5%) held a higher education degree, 51 (25.8%) had graduated from secondary education, and 9 (4.5%) had completed only primary education or vocational training without a formal graduation. Three individuals did not report their highest level of education.

When asked about their financial situation, 88 participants (44.6%) rated it as good or very good, 99 (50.3%) as adequate, and 10 (5.07%) as poor or very poor. Data were missing for three participants.

### 3.2. Changes in CVD Risk Estimation Accuracy Between 2023 and 2024

**Figure 2:**
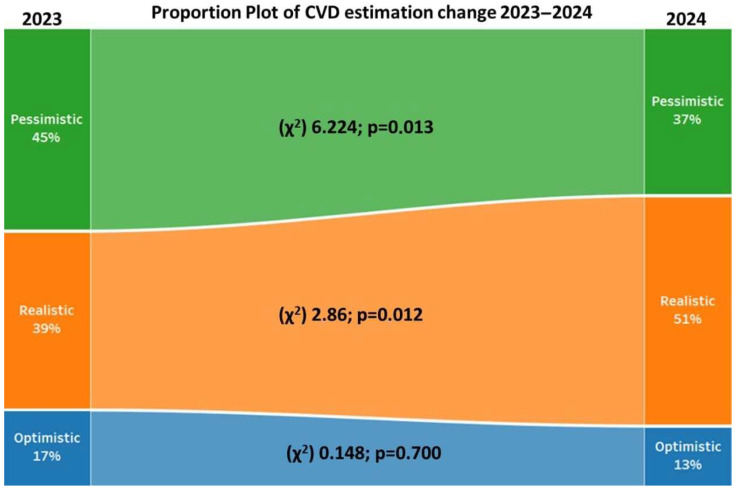
Changes in CVD estimation between 2023 and 2024.

In 2024, the proportion of realistic estimators significantly increased from 39.0% (*n* = 78) to 50.5% (*n* = 101) (χ^2^ = 6.286, *p* = 0.012), accompanied by a significant decrease in pessimists (χ^2^ = 6.224, *p* = 0.013), while the proportion of optimists remained unchanged. All results are presented in Table 1 and Table 2. The graphical presentation of the results is shown in Figure 2.

### 3.3. Comparison of Subjective Risk Estimation and Framingham Categories

We examined the distribution of individuals across different Framingham risk categories in 2024, according to their subjective perception of CV risk (Table 3). Among realistic estimators (*n* = 101), the majority (60.4%) were classified as low-risk, followed by 32.6% in the moderate-risk category and 6.9% in the high-risk category. Pessimistic estimators (*n* = 68) were predominantly classified as low-risk (89.7%), with a small proportion in the middle-risk category (10.3%) and none in the high-risk category. In contrast, optimistic estimators (*n* = 30) were evenly distributed between the moderate-risk (50%) and high-risk (50%) categories, with none in the low-risk category. This effectively capture the risk perception gap, particularly among pessimistic and optimistic estimators.

### 3.4. Association of Risk Communication Strategies and Estimation Accuracy

Table 4 presents the distribution of participants categorized by estimation accuracy across three different risk communication strategies. In the written-only format, which included the largest number of participants (*n* = 108), realists were the most prevalent (50%), followed by pessimists (35.2%) and optimists (14.8%). In the individual setting (*n* = 57), realists formed the largest group (43.8%), but the proportion of optimists (19.3%) was higher than in the written-only format group, while pessimists comprised 36.8% of the participants. The group with the smallest sample size (*n* = 34) had the highest proportion of realists (64.7%) and the lowest proportion of optimists (8.8%).

We found no association between risk communication strategies and estimation accuracy in 2024 (χ^2^ = 4.152; *p* = 0.386; Camer’s V = 0.102).

We also examined whether risk communication strategies were associated with changes in risk estimation accuracy. The accuracy of estimates remained unchanged for 72 participants, improved for 50, and was more accurate for 26 participants one year earlier. Among those who received only written feedback, 24.1% achieved improved estimation accuracy one year later. As shown in Table 5, no association was found between risk communication and changes in estimation accuracy (χ^2^ = 9.29; *p* = 0.153; Camer’s V = 0.154).

### 3.5. Association of Risk Communication Strategies and Medical Data

When examining risk communication strategies, we found that participants who received only written communication achieved significant reductions in body weight (*p* = 0.014), BMI (*p* = 0.042), systolic blood pressure (*p* = 0.035), and LDL levels (*p* = 0.001). In contrast, HDL levels increased significantly (*p* = 0.018).

Similar changes were observed among those who also received individual risk communication. In this group, body weight (*p* = 0.007), hip circumference (*p* = 0.009), BMI (*p* = 0.034), and LDL levels (*p* < 0.001) decreased significantly, while HDL levels increased (*p* = 0.003).

In contrast, only systolic blood pressure (*p* = 0.027) and fasting blood glucose (*p* = 0.013) showed significant reductions among participants who received group-based risk communication. Results are shown in Table 6.

## 4. Discussion

In this study, we investigated changes in CVD risk perception (perception gap) among participants in the Extended Budakalász Epidemiological Study between 2023 and 2024, focusing on the potential role of different risk communication strategies in these changes.

Our findings indicate a statistically significant improvement in the accuracy of CVD risk perception, primarily due to a decrease in pessimistic estimations. These results support our initial hypothesis and highlight the potential of targeted communication to align subjective and objective risk perceptions. However, the absence of differences among communication strategies limits conclusions about the relative effectiveness of specific approaches. Due to the quasi-randomized design, observed improvements cannot be attributed solely to the mode of risk communication, as unmeasured factors may have influenced the results. Consequently, these findings should be considered as indicative evidence. To our knowledge, no previous study with a comparable design has reported similar findings. From a clinical perspective, individuals who underestimate their CVD risk—despite being classified as moderate- or high-risk—warrant particular attention. Everett et al. (2016) [29] reported that 50% of hospitalized patients with coronary heart disease (CHD) perceived their risk of a future cardiac event as low, despite clinical evidence to the contrary. Contributing factors may include limited knowledge of risk factors, optimistic bias as a coping strategy, or downward social comparisons [10,30]. These findings can also be interpreted within the framework of the Health Belief Model [4], which suggests that individuals are more likely to engage in preventive health behaviors when they perceive themselves to be personally at risk. An accurate perception of cardiovascular risk thus serves as a motivational driver for adopting lifestyle changes and adhering to treatment. Although changes in perception alone do not guarantee improved clinical outcomes, they are considered a necessary prerequisite for sustained lifestyle modification and adherence to preventive strategies. Therefore, the observed improvements in perception accuracy, particularly when accompanied by favorable changes in objective risk factors, may have meaningful implications for long-term cardiovascular prevention [31,32].

Importantly, we did not observe statistically significant differences in estimation accuracy across communication strategies. Although participants receiving written-only feedback demonstrated improvements in both risk perception and several objective cardiovascular risk factors (Body weight, BMI, LDL, HDL) comparable to those receiving additional individual or group sessions, this finding should be interpreted with caution. The absence of true randomization and the potential influence of residual confounding limit causal inference. This general pattern of improvement is consistent with previous research, which has shown that even minimal interventions can shift risk perception. For example, studies within the NHS Health Check program in the UK indicate that providing personalized cardiovascular risk information increases the accuracy of risk estimation and enhances knowledge, compared to generic risk information [33]. In addition, a meta-analysis in the European Heart Journal found that structured risk information—regardless of delivery mode—can facilitate improved understanding and recalibration of perceived risk [21]. The same meta-analysis also confirmed that risk communication strategies are often associated with greater risk understanding and modest improvements in lifestyle markers [21].

The absence of statistically significant differences between communication strategies may reflect methodological limitations and limited statistical power, particularly in the group-based communication, which included fewer participants (*n* = 34 vs. *n* = 57 and *n* = 108). In addition, the interventions may not have differed sufficiently in intensity to produce detectable between-group effects. Furthermore, variability in intervention adherence represents an important source of heterogeneity: a substantial proportion of participants did not attend individual or group sessions, which may have attenuated potential differences between communication strategies.

Importantly, consistent improvements were observed across all groups in objective risk factors, suggesting that cardiovascular risk assessment combined with feedback—regardless of format—may have contributed to these changes. Although improvements in objective risk markers cannot be attributed solely to communication, they may partly reflect downstream effects of increased risk awareness, such as greater health consciousness, lifestyle changes, or treatment initiation. It is therefore likely that multiple, interacting pathways contributed to the observed improvements, with risk communication representing a key initiating factor.

Interestingly, in the group-based communication, no participants demonstrated a deterioration in risk estimation accuracy over time. Although this observation may point to a potential stabilizing effect of group interactions, it should be noted that it may have been driven by the small sample size and the exploratory nature of the analysis.

We employed the Framingham Risk Score, selected for its clinical familiarity, simplicity, and patient comprehensibility, to communicate CVD risk. Although newer tools such as SCORE2 are available, the Framingham Risk Score remains widely used in primary care and lends itself well to visual representation (e.g., bar charts), which can facilitate shared decision-making [19,23,24]. Previous studies have shown that CVD risk screening does not negatively affect mental health outcomes [33,34,35], supporting its broader application in clinical practice.

### 4.1. Implications for Practice

Our findings suggest that cardiovascular risk communication in primary care may not necessarily require resource-intensive interventions to influence patients’ perception of risk. In particular, written, personalized risk feedback appears to be a feasible and scalable approach for improving the accuracy of risk perception. Our implications should be interpreted in the context of the study design. Improvements in risk perception represent an important intermediate step in cardiovascular prevention, although their translation into sustained behavioral change and long-term clinical outcomes may vary and warrant further investigation. The effectiveness of written feedback may be attributed to personalized comparisons between general population-level results and individual participant data, as well as tailored recommendations.

In practical terms, this approach could be implemented by generating standardized risk reports during routine care and incorporating simple visual aids and tailored explanations. Although additional counseling may provide incremental benefits, it may not be essential for improving risk perception and selected clinical parameters. Effective communication should emphasize clarity, personalization, and patient engagement, and may benefit from reinforcement over time.

### 4.2. Strengths and Limitations

One of the main strengths of our study lies in its aim to develop a preventive protocol by examining risk communication strategies. This is particularly relevant given the absence of research data with a similar research design focused on correcting CVD perception bias. To our knowledge, such studies remain scarce, despite cardiovascular diseases representing a significant public health issue, and illness perception being an established determinant of health behavior.

However, our study has several limitations. Firstly, the study did not employ true randomization; although a systematic allocation procedure was used, it does not fully eliminate the possibility of residual selection bias, which is particularly relevant when interpreting the absence of differences between risk communication groups. Second, the overall sample size is relatively small from an epidemiological perspective, resulting in some of our group intervention samples being rather small (group intervention: *n* = 34). Third, intervention adherence was variable, with a substantial proportion of participants who did not attend individual and group sessions. This resulted in heterogeneous exposure to the risk communication and may have attenuated potential effects. The high proportion of married participants may bias the results, as marriage is a protective factor for health. Also, it should be noted that our sample was well educated (69.5% with higher education), which further limits the generalizability of our results. Furthermore, several of our variables were ordinal or categorical, limiting the applicability of specific statistical tests during data analysis. In addition, no correction for multiple comparisons (e.g., Bonferroni adjustment) was applied in the analysis of multiple clinical parameters, increasing the risk of Type I error, therefore some statistically significant findings should be interpreted with caution Although single-item measures of subjective risk perception are widely accepted and used in cardiovascular research, they may not fully capture the complexity of the construct, which could be considered a limitation of the present study.

Given these limitations, further research—preferably using randomized designs and larger, more diverse samples—is needed to confirm these findings and clarify causal relationships.

### 4.3. Future Directions

Although the improvement in estimation accuracy after one year is promising, questions remain about risk communication strategies and the long-term sustainability of this effect. Risk perception is dynamic and may diminish without continued reinforcement over time. Future studies should examine whether periodic risk communication or follow-up sessions are necessary to maintain accurate perceptions and support sustained behavioral change. Integrating structured risk feedback into regular clinical encounters could be a potential strategy to address this issue.

We consider it necessary to increase the sample size within the risk groups to conduct an effectiveness analysis, thereby providing a clearer understanding of the most effective risk communication strategies for the clinically most relevant group, namely optimists.

## 5. Conclusions

Our study demonstrates that a comprehensive cardiovascular risk prevention program in a primary care setting can significantly enhance individuals’ ability to accurately perceive their own risk. Between 2023 and 2024, the proportion of participants with accurate risk estimations increased, while pessimistic perceptions decreased, highlighting the potential of structured communication—even in the absence of a single superior method. Accurate risk perception is clinically important, as it may motivate adherence to preventive behaviors and lifestyle modifications, particularly among those at moderate or high objective risk. Although the additional benefit of individualized consultation was modest, the results suggest that only written, clear, comprehensible, and contextually relevant information may be sufficient to initiate meaningful changes in cardiovascular risk factors. These insights highlight the potential for scalable, cost-effective strategies to enhance preventive care in community settings.

## Figures and Tables

**Table 1:** Distribution of risk estimation accuracy in 2023 and in 2024.

		Subjective Risk Estimation 2024			
		Realist	Pessimist	Optimist	Total
Subjective risk estimation 2023	Realist	51	17	10	78
		65.40%	21.80%	12.80%	100%
		50.50%	24.60%	33.30%	39%
	Pessimist	37	50	2	89
		41.60%	56.20%	2.20%	100%
		36.60%	72.50%	6.70%	44.5%
	Optimist	13	2	18	33
		39.40%	6.10%	54.50%	100%
		12.90%	2.90%	60%	16.5%
	Total	101	69	30	200
		50.50%	34.5%	15%	100%
		100%	100%	100%	100%

**Table 2:** Statistical analysis of changes in CVD risk estimation accuracy between 2023 and 2024.

	2023	2024		χ^2^	*p*
Realistic rate changes (78 → 101)		correct estimation	incorrect estimation	6.286	0.012
	correct estimation	51	27		
	incorrect estimation	50	72		
Pessimist rate changes (89 → 69)		other estimation	over-estimation	6.224	0.013
	other estimation	92	19		
	overestimation	39	50		
Optimist rate changes (33 → 30)		other estimation	under-estimation	0.148	0.700
	other estimation	155	12		
	underestimation	15	18		

**Table 3:** Distribution of subjective risk estimation and Framingham categories.

		Objective Risk Assessment: Framingham Risk Category 2024			
		Low Risk	Middle Risk	High Risk	Total
Subjective risk estimation 2024	Realist	61 (60.4%)	33 (32.6%)	7 (6.9%)	101 (100%)
	Pessimist	61 (89.7%)	7 (10.3%)	0 (0%)	68 (100%)
	Optimist	0 (0%)	15 (50%)	15 (50%)	30 (100%)

**Table 4:** Distribution of estimation accuracy types across risk communication formats.

		Subjective Risk Estimation 2024				
		Realist	Pessimist	Optimist	Total	*χ*^2^ (*p*)
Risk Communication Strategy	Written-only	54 (50%)	38 (35.2%)	16 (14.8%)	108 (100%)	4.152 (0.386)
	Written + Individual	25 (43.8%)	21 (36.8%)	11 (19.29%)	57 (100%)	
	Written + Group	22 (64.7%)	9 (26.5%)	3 (8.8%)	34 (100%)	
	Total	101	68	30	199	

**Table 5:** Distribution of changes in estimation accuracy across risk communication strategies.

		Changes in Estimation Accuracy				
		Stagnation	Improvement	Deterioration	Total	*χ*^2^ (*p*)
Risk Communication Strategy	Written-only	67 (62.0%)	26 (24.1%)	15 (13.9%)	108 (100%)	9.29 (0.153)
	Written + Individual	35 (61.4%)	11 (19.3%)	11 (19.3%)	57 (100%)	
	Written + Group	21 (61.8%)	13 (38.2%)	0 (0%)	34 (100%)	
	Total	72	50	26	199	

**Table 6:** Medical data across the risk communication groups between 2023 and 2024.

	CVD Risk Communication								
Variables	Written-Only			Written + Individual			Written + Group		
	Z	*p*	Effect size	Z	*p*	Effect size	Z	*p*	Effect size
Framingham score	−0.198	0.843		−0.139	0.890		−1.421	0.155	
Body weight	−2.469	0.014	0.237	−2.702	0.007	0.358	−0.974	0.330	
Hip circumference	−1.474	0.141		−2.631	0.009	0.365	−0.194	0.846	
Stomach circumference	−1.117	0.264		−0.612	0.541		−1.029	0.303	
BMI	−2.038	0.042	0.196	−2.121	0.034	0.281	−0.043	0.966	
RRSys	−2.112	0.035	0.204	−0.388	0.698		−2.217	0.027	0.380
Cholesterol	−0.24	0.810		−1.069	0.285		−0.465	0.642	
Triglyceride	−0.152	0.879		−0.110	0.912		−0.718	0.473	
LDL	−3.407	0.001	0.374	−3.810	0.000	0.534	−0.408	0.683	
HDL	−2.373	0.018	0.228	−2.980	0.003	0.395	−1.132	0.258	
Glucose	−0.045	0.964		−0.575	0.565		−2.490	0.013	0.433
HbA1c%	−1.803	0.071		−1.292	0.196		−0.608	0.543	

Note: BMI = Body Mass Index; RRSys = Systolic blood pressure; LDL = Low-density lipoprotein; HDL = High-density lipoprotein; HbA1c% = Hemoglobin A1c perce.

## Data Availability

The datasets generated and/or analyzed during the current study are not publicly available due to the protection of medical information but are available from the corresponding author on reasonable request.

## Supplementary Materials

The following supporting information can be downloaded at: https://www.mdpi.com/article/10.3390/healthcare14091229/s1, Document S1: Written Risk Communication example.

## Abbreviations

The following abbreviations are used in this manuscript:

CVD	Cardiovascular Disease
CV	Cardiovascular
WHO	World Health Organization
ESC	European Society of Cardiology
