# Supplementary material for: From Misperception to Prevention: Improving Cardiovascular Health and Risk Perception Through Risk Communication in Hungary
Source: Healthcare (Basel). 2026 May 3;14(9):1229. doi: 10.3390/healthcare14091229 (PMC13164021; doi:10.3390/healthcare14091229)
Supplement: Supplementary file 1 [file healthcare-14-01229-s001.zip › healthcare-4206960-supplementary.pdf]

Dear……!

Thank you for participating in our research project entitled "Extended Budakalász Epidemiological Study: Health Behavior - Measuring Willingness to Change Following Cardiovascular Risk Assessment" and our lifestyle change program!

Getting a more accurate picture of your cardiovascular risk status will help you make more informed choices from the free health improvement programs starting in April 2023 (group exercise classes, dietary counselling, recipe exchange club, and club sessions supporting health behavior change). The first step in the process was to assess your cardiovascular risk, the results of which are shown below.

During the examination, we measured parameters that are important and influential in terms of cardiovascular risk.

Below, you will receive feedback on the laboratory and other parameters that contribute to your cardiovascular condition as risk factors. The limit values are the internationally recommended limit values. These may differ in the case of other existing non-infectious diseases! If you have such a disease, please consult your family doctor for more detailed information about your condition.

Summarized Cardiovascular Risk

Below you will find two types of risk assessment, **the Framingham and SCORE2 scores**, expressed as percentages. Next to each, you will find a brief explanation of what this means for you. The two risk assessment methods take different parameters into account, and both results are important in terms of cardiovascular risk.

Your risk of cardiovascular disease based on the Framingham scale: 4.3% (Source: Framingham Heart Study)

This means that if you continue your current lifestyle, you have a **4.3** percent chance of developing some form of cardiovascular disease in the next 10 years. This is an estimate, which means that out of 100 people with a lifestyle and condition similar to yours, approximately 4 will develop cardiovascular problems within 10 years.

Framingham risk percentages group classification		
**0-9%: low**	**10-19%: middle**	**20% or more: high**

Your risk classification according to the SCORE2 method: **2.3%**, which in your case means low risk.

(Source: esccardio.org)

Other physical examination results

At this point, you will find the values recorded by our colleagues in the baseline assessment, and below these, you will find a summary of the relevant recommendations of domestic and international professional organizations regarding target values to help you interpret the results.

Blood pressure: 135/78 Hgmm
Body weight: 66.8 kg
Height: 157 cm
BMI: 27.1
FINDRISK (risk score of type-2 diabetes): 11

Help interpreting the results

These target values are optimal values that may be modified by certain chronic and acute conditions. Please consult your family doctor for an individual assessment of your results.

CLASSIFICATION OF BLOOD PRESSURE VALUES
**Below 120 Hgmm:** We talk about optimal blood pressure when blood pressure does not exceed 120/80 mmHg.
**Between 120 and 129 Hgmm:** We still consider it normal if it is higher than this, but below 130/85 mmHg.
**Between 130 and 139 Hgmm**: We consider blood pressure to be elevated at systolic values between 130-139 mmHg and/or diastolic values between 85-89 mmHg.
**Above 140 Hgmm:** High blood pressure begins at a systolic value of 140 mmHg and/or a diastolic value of 90 mmHg.
(Source: Hungarian Hypertension Society)

CLASSIFICATION OF BODY-MASS INDEX (BMI)
**Below 18,5:** You are malnourished, which poses a health risk. You need to increase your energy intake. If you do not gain weight proportionally, be sure to consult your family doctor!
**Between 18,5 and 24,9:** Keep up the good work, maintain your weight!
**Between 25 and 29,9:** You are overweight, which poses a health risk. Review your calorie intake, diet, and physical activity. If you need support, seek professional help!
**Between 30 and 34,9:** You are obese, which carries increased health risks, e.g., a higher risk of developing type 2 diabetes and cardiovascular disease. Consult your family doctor for further action.
**Above 35:** You are severely obese, which poses extreme health risks, e.g., a very high risk of developing type 2 diabetes and cardiovascular disease. Please consult your family doctor as soon as possible to discuss further steps.
**Note:** The evaluation is of limited use for patients who lead a particularly sedentary lifestyle or athletes who engage in sports that involve significant muscle mass gain.

FINDRISK (type 2 diabetes) - source: FINDRISK questionnaire

Less than 7 points: low risk. It is estimated that 1 in 100 people will develop diabetes.

7–11 points: slightly increased risk. It is estimated that 1 in 25 people will develop diabetes.

12–14 points: increased risk. It is estimated that 1 in 6 people will develop diabetes.

15–20 points: high risk. It is estimated that 1 in 3 people will develop diabetes.

More than 20 points: very high risk. It is estimated that 1 in 2 people will develop diabetes within 10 years.

If you scored less than 7 points, you have a low risk of developing type 2 diabetes. If you scored 7 points or higher, you need to pay closer attention to your lifestyle and reduce the risks that you can influence.

LABORATORY RESULTS

Here you will find the laboratory results that we have recorded in our system based on the laboratory findings you provided. Below these, you will find a brief summary of the normal ranges according to domestic and international recommendations:

Total cholesterol: 3.0 mmol/L
Triglyceride: 0.75 mmol/L
HDL: 1.23 mmol/L
LDL: 3 mmol/L
Hba1c: 5.8 mmol/L
Fasting blood sugar level: 5.6 mmol/L

Help interpreting the results

CLASSIFICATION OF BLOOD FAT LEVELS

Total cholesterol below 5.2, triglycerides below 2.2, HDL above 1.6, LDL below 2.6: The optimal range for blood lipid levels. Lifestyle, proper nutrition, and regular exercise help maintain these levels.

Total cholesterol 5.2-6.2, triglycerides 2.3-5.6, HDL 1.6-1.1, LDL 2.6-4.1: Elevated blood lipid levels. By changing your lifestyle and introducing a diet, their levels can be brought into the optimal range.

Total cholesterol >6.2, triglycerides >5.6, HDL <1.1, LDL >4.1: High blood lipid levels. These significantly increase the risk of cardiovascular disease. Medical treatment and regular monitoring are recommended.

Normal fasting blood sugar level
By keeping your blood sugar level as close to normal as possible, you can prevent many complications that could affect your nerves and blood vessels later on. In addition, proper control will also help you improve your quality of life. Normal blood sugar levels are generally between 4 and 6 mmol/l before meals.<br>Measured in a laboratory, ≤ 6.0 mmol/l, and during self-monitoring, ≤ 5.5 mmol/l is considered normal. Since blood sugar levels rise after meals, blood sugar values before and after meals are different. (source: http://www.diabet.hu/info.aspx?web_id=&sp=167, Hungarian Diabetes Association)

GLYCATED HEMOGLOBIN (HbA1c) RATIO
This is a value that indicates the average blood sugar level over the past three months. In healthy people, a value below 6.5% is normal; if you are being treated for diabetes, the target range is below 7%.

PSYCHOLOGICAL TEST RESULTS

Here you will find important results for assessing your cardiovascular risk.

Mood characteristics (Source: BDI):

You can complete the questionnaire at the link below; it will take no more than two minutes. Please provide your email address when completing the questionnaire. We will send feedback on your mood characteristics electronically to the email address you provide when completing the questionnaire.

Internet address: shorturl.at/xLVXZ

Perceived stress:

Based on your answers, your stress level is average, similar to that of the majority of the Hungarian population. It is important to continue paying attention to this in order to maintain your health. It is worth maintaining this level and preventing it from rising.

Recommendations for reducing cardiovascular risk

How can cardiovascular risk be reduced?

Influencing and modifying lifestyle:
(Source: https://www.doki.net/tarsasag/kardiologia_paciens/hirek.aspx?nid=107220)

- Weight control: according to professional recommendations, losing 5-10 percent of our body weight already reduces the risk of developing cardiovascular disease;

- Regular physical exercise: professional recommendations prescribe 150 minutes of moderate-intensity physical exercise per week;

- Heart-healthy diet: eat foods that are rich in polyunsaturated and omega-3 fatty acids, such as oily fish with vegetables and fruit. These are healthy and also reduce the risk of cardiovascular disease. Reducing your intake of meat products, salt, saturated fatty acids, and added sugar has the same effect.

- Quitting smoking: Smoking is a major risk factor for almost all cardiovascular diseases. It is best not to start smoking at all, thus avoiding addiction, but if you already smoke, it is recommended that you quit as soon as possible. Once you take steps in this direction, the harmful effects of smoking on the cardiovascular system will also decrease significantly.

The influence of stress and how to modify it:

Long-term, chronic stress can have a detrimental effect on the immune system and overall health. If you feel that you live a stressful life and your scores indicate this, it is important to pay more attention to this issue so that you can enjoy more years of good health. It is worth knowing that even if you cannot eliminate all sources of stress from your life, you can actively work to reduce your stress levels! For example, spending quality time with family and friends, physical activity (jogging, walking, etc.), relaxing in nature, engaging in leisure activities or even trying new ones, and learning relaxation techniques can be effective. Try to incorporate as many of these as possible into your daily routine!

- Our mood and stress levels also affect our health behavior. Therefore, consciously influencing and reducing both our mood and stress levels also has an impact on the health of our heart.

- If you feel hopeless or extremely depressed, we recommend that you explore the causes further, for which you may seek professional help. Contact your family doctor, who will refer you to a specialist who can help you during this period.
